# Regulatory T Cells in Melanoma Revisited by a Computational Clustering of FOXP3^+^ T Cell Subpopulations

**DOI:** 10.4049/jimmunol.1402695

**Published:** 2016-02-10

**Authors:** Hiroko Fujii, Julie Josse, Miki Tanioka, Yoshiki Miyachi, François Husson, Masahiro Ono

**Affiliations:** * Department of Dermatology, Graduate School of Medicine, Kyoto University, Kyoto 606-8501, Japan;; † Laboratoire de Mathématiques Appliquées, Agrocampus Ouest, 35042 Rennes Cedex, France;; ‡ Department of Life Sciences, Faculty of Natural Sciences, Imperial College London, London SW7 2AZ, United Kingdom; and; § Immunobiology, University College London Institute of Child Health, London WC1N 1EH, United Kingdom

## Abstract

CD4^+^ T cells that express the transcription factor FOXP3 (FOXP3^+^ T cells) are commonly regarded as immunosuppressive regulatory T cells (Tregs). FOXP3^+^ T cells are reported to be increased in tumor-bearing patients or animals and are considered to suppress antitumor immunity, but the evidence is often contradictory. In addition, accumulating evidence indicates that FOXP3 is induced by antigenic stimulation and that some non-Treg FOXP3^+^ T cells, especially memory-phenotype FOXP3^low^ cells, produce proinflammatory cytokines. Accordingly, the subclassification of FOXP3^+^ T cells is fundamental for revealing the significance of FOXP3^+^ T cells in tumor immunity, but the arbitrariness and complexity of manual gating have complicated the issue. In this article, we report a computational method to automatically identify and classify FOXP3^+^ T cells into subsets using clustering algorithms. By analyzing flow cytometric data of melanoma patients, the proposed method showed that the FOXP3^+^ subpopulation that had relatively high FOXP3, CD45RO, and CD25 expressions was increased in melanoma patients, whereas manual gating did not produce significant results on the FOXP3^+^ subpopulations. Interestingly, the computationally identified FOXP3^+^ subpopulation included not only classical FOXP3^high^ Tregs, but also memory-phenotype FOXP3^low^ cells by manual gating. Furthermore, the proposed method successfully analyzed an independent data set, showing that the same FOXP3^+^ subpopulation was increased in melanoma patients, validating the method. Collectively, the proposed method successfully captured an important feature of melanoma without relying on the existing criteria of FOXP3^+^ T cells, revealing a hidden association between the T cell profile and melanoma, and providing new insights into FOXP3^+^ T cells and Tregs.

## Introduction

Regulatory T cells (Tregs) are defined as the immunosuppressive T cells that suppress the activities of other T cells through undefined mechanisms, and they are identified by the transcription factor FOXP3 (1). Although Tregs are reported to be increased in tumor-bearing patients or animals, and thereby suppress antitumor immunity (2–4), the evidence is in fact mixed (5): the increase of FOXP3^+^ T cells is associated with poor prognosis in hepatocellular cancer (6), whereas it is related to good prognosis in colorectal cancer (7). The discrepancy may be explained by that FOXP3^+^ T cells include not only regulatory but also non-Tregs that produce proinflammatory cytokines (8). In fact, accumulating evidence indicates that FOXP3 is not the definitive marker for the immunosuppressive T cells in humans. The expression of FOXP3 can be induced in naive T cells by conventional anti-CD3 stimulation (9, 10). In addition, some FOXP3^+^ T cells, especially memory-phenotype CD45RO^+^FOXP3^low^ cells, produce effector cytokines and are not suppressive by an in vitro assay, suggesting that they are enriched with effector and activated T cells (9).

Accordingly, the subclassification of FOXP3^+^ T cells has been a major issue in human Treg research (8, 9, 11–17). It was proposed that FOXP3^+^ T cells could be classified into three functionally different subpopulations: CD45RO^+^ (equivalent to CD45RA^−^) FOXP3^high^ T cells as classical Tregs with suppressive activity (9, 11), CD45RO^−^ (or CD45RA^+^) FOXP3^low^ naive Tregs (9, 12, 13), and FOXP3^low^CD45RO^+^ non-Tregs (9, 14, 15). This classification has been used to analyze FOXP3^+^ T cells in autoimmune diseases and cancers (8, 9, 16, 17). Unfortunately, however, the definition of FOXP3^+^ subpopulations varies between studies, complicating the problem (18). Meanwhile, recently, Abbas et al. (19) proposed not to use new terms for Treg subpopulations until a new population has been extensively demonstrated to be unique, distinct from other populations and stable, because it is likely to lead to more confusion and the further jargonizing of immunology. This opinion, however, ignores the fact that a clustering (classification) approach, whether manual or automatic gating, is indispensable for summarizing and analyzing flow cytometric data, and thereby relating immunological profiles to biological response or disease status (20, 21).

Currently in experimental immunology, any cellular populations, including FOXP3^+^ T cells, are almost always identified and analyzed by *manual gating*, which is a process of identifying a cluster of cells by manually drawing regions, or *gates*, in two-dimensional graphical representations of the data (22–24). Obviously, manual gating is subjective and cannot fully use multidimensional flow cytometry data; therefore, the automatic gating using clustering methods has become an active research area of bioinformatics over the past several years (23, 25). In fact, a preceding study proposed a computational approach to identify a Treg population, precisely CD4^+^CD25^+^DR^+^FOXP3^+^ cells (26), but it did not address the immunological significance of the approach and that of the identified Treg population.

In this study, we aimed to establish a computational approach to identify and classify FOXP3^+^ T cells into subpopulations, addressing the immunological and clinical significance of the method, and thereby to revisit the fundamental subclassification of FOXP3^+^ T cells. To establish the method, we used a data set of PBMCs from melanoma patients and healthy controls (HCs), which previously identified that total FOXP3^+^ T cells increased in melanoma patients, and that FOXP3^low^ naive Tregs and FOXP3^low^ non-Tregs increased as the stage progressed (8). Furthermore, in this study, we have newly obtained a flow cytometric data set of FOXP3^+^ T cells from melanoma patients and HCs (designated as the second data set), to address the efficiency of the proposed method. Thus, we first show the clinical and immunological significance of a data-oriented clustering approach to the subclassification of the FOXP3^+^ T cells.

## Materials and Methods

### Patient samples

All PBMC data sets were obtained from patients with malignant melanoma who were treated in the Department of Dermatology, Kyoto University Hospital. The first data set analyzed 23 individuals by FACSCalibur (BD Biosciences) (8). The second data set analyzed 19 individuals by LSR Fortessa (BD Biosciences). The patients’ characteristics in the second data set are summarized in Table I. We also obtained data from age- and sex-matched HCs (first data set, *n* = 28; second data set, *n* = 15). This study was approved by the Medical Ethics Committee of Kyoto University and was conducted in accordance with the principles of the Declaration of Helsinki. All participants provided written informed consent.

### Flow cytometric analysis

PBMCs were isolated with Ficoll–Isopaque (Lymphoprep; Axis-Shield, Oslo, Norway) gradient centrifugation. All cells were freshly stained with the following mAbs and analyzed promptly as previously described (9): FITC-conjugated anti-CD45RO (UCHL1; BD Biosciences); PE-conjugated anti-CD25 (M-A251; BD Biosciences); PerCP-Cy5.5–conjugated anti-CD4 (SK3; BD Biosciences); and biotinylated anti-FOXP3 (236A/E7; eBioscience, San Diego, CA) and allophycocyanin-streptavidin (BD Biosciences). The second data set (Table I) was obtained by LSR Fortessa (BD Biosciences), using the following settings for voltage: FSC-A (319), SSC-A (335), FL1-A (CD45RO, 582), FL2-A (CD25, 478), FL3-A (CD4, 742), and FL4-A (FOXP3, 676). FlowJo (Tree Star) was used for manual gating.

### Automatic gating of FOXP3^+^ T cell subpopulations

For data preprocessing, boundary values were removed [i.e., ≥1000 or <100 (forward scatter [FSC]), ≥1000 (side scatter [SSC]), ≥4 or <0.3 (fluorescence channels, logged) in the case of analog data; ≥800 or <100 (FSC), ≥1000 (SSC), and ≥3.5 or <0.1 (fluorescence channels, logged) in the case of digital data], because they were considered meaningless events representing cellular debris or large nonlymphocytes, or noise (27). Subsequently, all fluorescence data were log-transformed, and each variable was normalized by the standardized scaling. In the established classification method (the HKK clustering [see the section headed *Automatic classification of the three FOXP3^+^ T cell subpopulations*]), FOXP3^+^ T cell subpopulations were identified by the following three steps: first, CD4^+^ T cells were clustered by a high-dimensional data clustering (HDDC) function, *hddc*, of a CRAN package, *HDclassif* (28) using FSC, SSC, and CD4 (*k* = 3). Second, FOXP3^+^ T cells were clustered by a k-means clustering of FOXP3 values using kmeans of a CRAN package, *Stats* (29), and the cluster containing the centroid with the highest FOXP3 value was designated as FOXP3^+^ T cells. The number of clusters (*k* = 3) was determined by examining the bar plot of the loss variability (30), and also taking into account the identification of the FOXP3^+^ cluster that has higher FOXP3 values than the FOXP3^−^ cloud. Third, finally, FOXP3^+^ T cell subpopulations were identified by a k-means clustering, using CD45RO, CD25, and FOXP3 with *k =* 3, and subsequently assigned to the Effector-Treg–like, Naive-Treg–like, and Non-treg–like clusters as follows: 1) compute the centroid of each cluster and designate the cluster containing the centroid with the highest value for FOXP3 as effector-Treg–like; and 2) among the two other clusters, the one with the smallest value for CD45RO as naive-Treg–like and the last one as non-treg–like. All computational analyses were done using a laptop with an Intel Core i5-3360 M CPU - 2.80 GHz or a Mac desktop with 3.5 GHz Intel Core i5, OS10.10.4.

### Statistical analysis

A Mann–Whitney–Wilcoxon test was used for analyzing two groups, testing the null hypothesis that the two statuses (i.e., HC or melanoma) have equal medians. A Kruskal–Wallis test, a nonparametric alternative to ANOVA, was used for analyzing more than two groups, testing the null hypothesis that the medians are equal across the groups (HC and disease stages), followed by pairwise comparisons using a Mann–Whitney–Wilcoxon test. The *p* values were adjusted by a Bonferroni procedure for multiple comparisons in all the analyses.

## Results

### Manual gating approach to FOXP3^+^ T cell subpopulations

**FIGURE 1.**
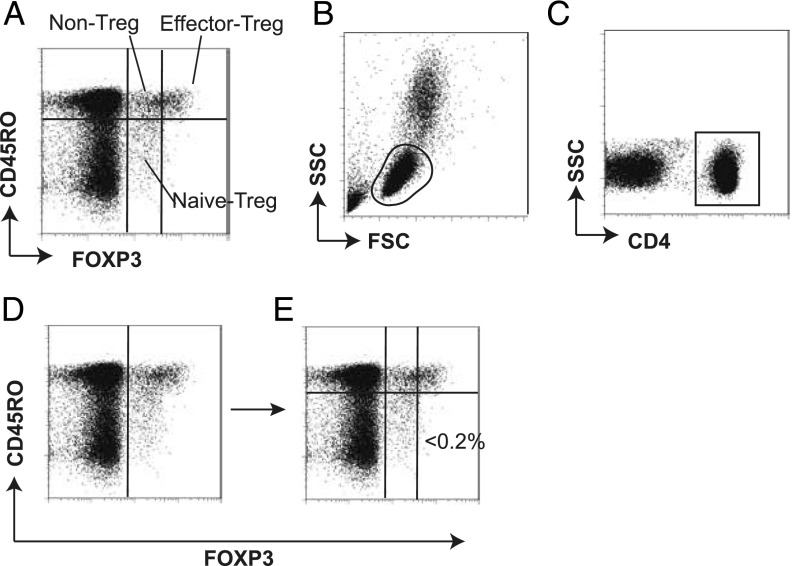
Manual gating approach to classify the FOXP3^+^ T cell subpopulations. The manual gating approach to identify and classify FOXP3^+^ T cell subpopulations is depicted. (**A**) Representative flow cytometric data showing the three subsets of FOXP3^+^ T cells in PBMCs: CD45RO^+^FOXP3^high^ effector Treg, CD45RO^−^FOXP3^low^ naive Treg, and CD45RO^+^FOXP3^low^ non-Treg. These subpopulations are classified in a sequential manner using the following four gates: (**B**) the lymphocyte gate on the window displaying FSC and SSC, (**C**) the CD4^+^ gate using CD4 and SSC, (**D**) the FOXP3^+^ gate using FOXP3 and CD45RO, and (**E**) the FOXP3^+^ T cell subpopulation gate using FOXP3 and CD45RO. The level of FOXP3^high^ and FOXP3^low^ cells was determined so that CD45RO^−^FOXP3^high^ cells were <0.2% of CD4^+^ T cells.

**FIGURE 2.**
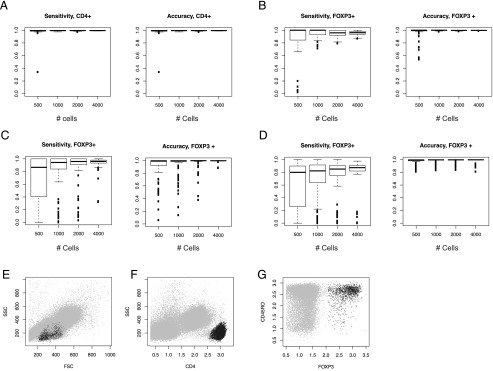
Automatic gating of FOXP3^+^CD4^+^ T cells. Three clustering methods were compared for identifying FOXP3^+^CD4^+^ T cells: CD4^+^ T cell selection by HDDC, followed by FOXP3^+^ T cell selection by k-means (HK clustering); CD4^+^ T cell selection by HDDC, followed by FOXP3^+^ T cell selection by HDDC (HH clustering); and FOXP3^+^CD4^+^ T cell selection by one step-HDDC (one-step H clustering). Various random number seeds were used to resample events from flow cytometric data, and resampling was repeated 100 times for each random number seed, to address the robustness and efficiency of the three clustering methods. Sensitivities and accuracies were calculated by assuming that the manual gating provides a gold standard. (**A**–**C**) Sensitivities and accuracies of HK and HH: (A) sensitivities and accuracies for identifying CD4^+^ T cells by HDDC (shared by HK and HH). (B and C) Sensitivities and accuracies of (B) k-means and (C) HDDC for identifying FOXP3^+^ T cells from the identified CD4^+^ T cell cluster (HK and HH, respectively). (**D**) Sensitivities and accuracies of HDDC for identifying FOXP3^+^ T cells from all cells (one-step H). (**E**–**G**) Representative plots of automatically gated FOXP3^+^CD4^+^ T cells by the HK clustering method for (E and F) CD4^+^ T cells and (G) FOXP3^+^ T cells. The clustered cells are shown by black dots.

**FIGURE 3.**
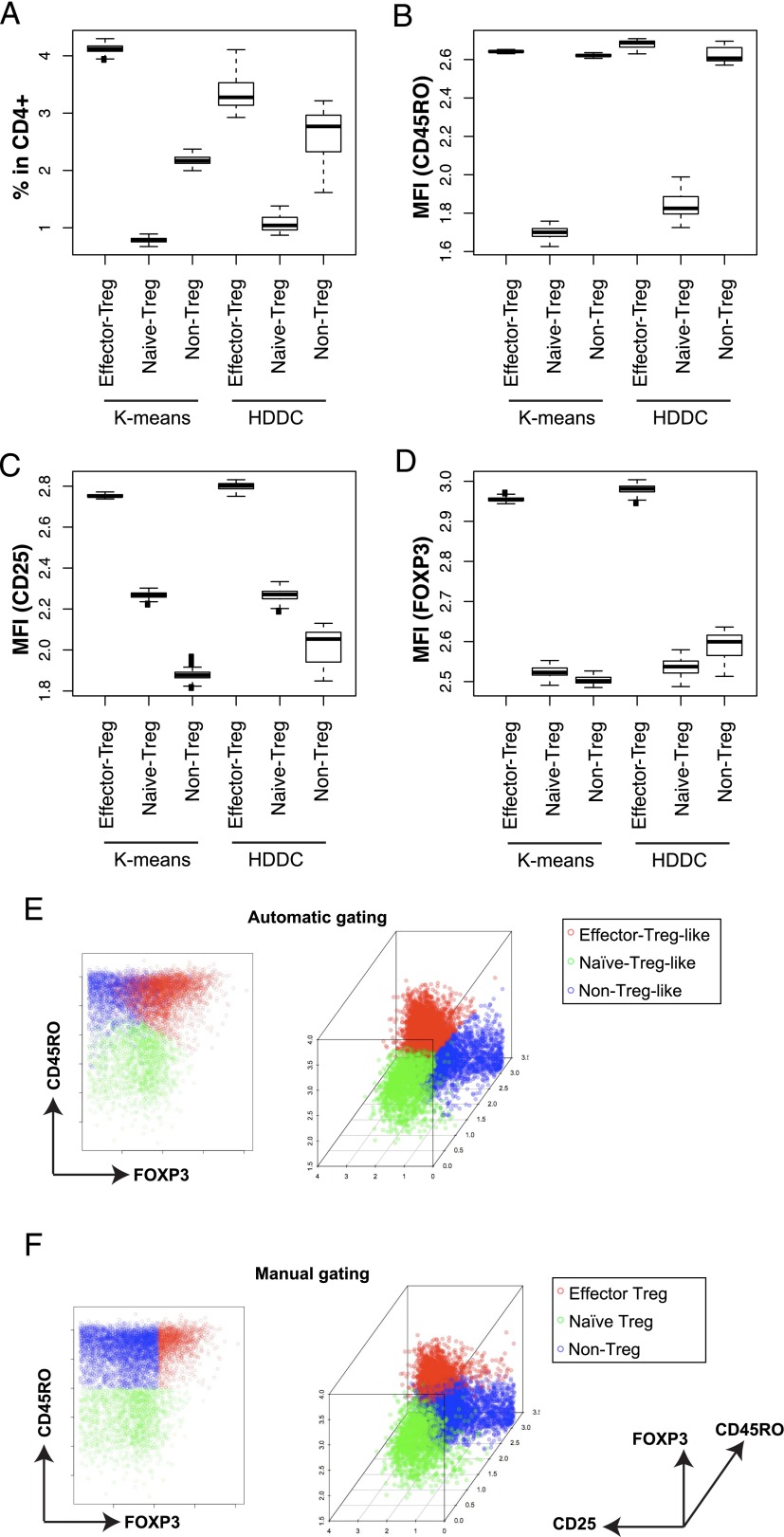
Automatic clustering of FOXP3^+^ CD4^+^ T cell subpopulations. K-means and HDDC were used for classifying the computationally clustered FOXP3^+^ T cells into three subpopulations, and we compared them for stability using a resampling approach, which was repeated 100 times. (**A**–**D**) Box plots showing (A) the percentages and the mean fluorescence intensities (MFI) of (B) CD45RO, (C) CD25, or (D) FOXP3 of each FOXP3^+^ T cell subcluster in CD4^+^ T cells by either k-means or HDDC in the 100 resampled samples (i.e., HKK or HKH, respectively). (**E** and **F**) Representative plots of FOXP3^+^ T cell subpopulations (E) by the HKK clustering (automatic) and (F) by manual gating.

This section shows how the standard approach, manual gating, identified and classified FOXP3^+^ T cells into three subsets: CD45RO^+^FOXP3^high^ effector (memory) Treg (effector-Treg population hereafter), CD45RO^−^FOXP3^low^ naive Treg (naive-Treg population), and CD45RO^+^FOXP3^low^ non-Tregs (non-Treg population) (8, 9) (Fig. 1A). These subpopulations were identified in a sequential manner using the following four gates: 1) the lymphocyte gate using FSC and SSC (Fig. 1B); 2) the CD4^+^ gate using CD4 and SSC (Fig. 1C); 3) the FOXP3^+^ gate using FOXP3 and CD45RO to visually identify a groove of the FOXP3 distribution (Fig. 1D); and 4) the FOXP3/CD45RO gate to identify the FOXP3^+^ T cell subpopulations (Fig. 1E). The level of FOXP3 by which FOXP3^high^ and FOXP3^low^ cells were separated was determined so that CD45RO^−^FOXP3^high^ cells were <0.2% of CD4^+^ lymphocytes using HCs (Fig. 1E).

### Data-oriented clustering (automatic gating) of FOXP3^+^ T cell subpopulations

We aimed to establish an automated clustering method for identifying FOXP3^+^ T cells and classifying them into three subpopulations, and thereby to revisit the immunological significance of the FOXP3^+^ T cell classification. Importantly, there is no major controversy regarding the identification of the total FOXP3^+^ CD4^+^ T cells, but there are multiple ways to classify FOXP3^+^ T cells, which we aimed to address in this study. Thus, we used the following approach to identify the FOXP3^+^ T cell subpopulations: 1) to identify FOXP3^+^CD4^+^ T cells; and 2) to classify FOXP3^+^CD4^+^ T cells into three subpopulations without using the manual gating strategy. We used the flow cytometric data set in our previous report, which was obtained by a FACSCalibur (8) (the first data set), to establish a clustering method.

#### Automatic gating of FOXP3^+^ CD4^+^ T cells.

The aim of this step is to efficiently and robustly identify FOXP3^+^ CD4^+^ T cells as described earlier. CD4^+^ cells are distinct from CD4^−^ cells in the space of FSC, SSC, and CD4 (Fig. 1B, 1C), whereas the distribution of FOXP3 expression is continuous in CD4^+^ T cells (Fig. 1D). In fact, a preliminary analysis showed that CD4^+^ T cells were efficiently identified using a model of HDDC (28), but not by a common clustering method, k-means. HDDC is based on Gaussian mixtures with restricted covariance matrices (28) and can efficiently identify elliptic populations such as CD4^+^ T cells in the space of FSC, SSC, and CD4. In contrast, FOXP3^+^ cells are not as discrete as CD4^+^ T cells, and it was not obvious what method was suitable.

Thus, we compared different combinations of the clustering methods using a resampling approach, addressing the sensitivity and the accuracy of the methods. In this study, we compared the following methods: 1) HK clustering: CD4^+^ T cell selection by HDDC, followed by FOXP3^+^ T cell selection by k-means; 2) HH clustering: CD4^+^ T cell selection by HDDC, followed by FOXP3^+^ T cell selection by HDDC; and 3) one-step H clustering: FOXP3^+^CD4^+^ T cell selection by one-step HDDC. Using several random number seeds, the HK clustering showed the highest sensitivities and accuracies across different cell numbers compared with the other methods (Fig. 2).

#### Automatic classification of the three FOXP3^+^ T cell subpopulations.

Next, we aimed to establish a method that subclassifies the FOXP3^+^CD4^+^ T cells into three subpopulations without relying on the manual gating criteria, and thereby to readdress the significance of FOXP3^+^CD4^+^ T cell subpopulations. We compared k-means and HDDC (*k* = 3; designated as HKK and HKH clustering methods, respectively) by a resampling approach, to identify a method that consistently assigns similar cells to each cluster, using CD45RO, CD25, and FOXP3. The resampling experiment showed that the HKK clustering had smaller variations in both the percentage and the mean fluorescence intensities of CD45RO, CD25, and FOXP3 of each subpopulation than the HKH clustering (Fig. 3A–D). Thus, the HKK clustering has been chosen as the method for identifying and classifying the FOXP3^+^ T cell subpopulations.

The three clusters identified by the HKK clustering were partially overlapped with the three subpopulations identified by manual gating. To make the clusters and the subpopulations comparable, the cluster with the highest FOXP3 expressions was designated as effector-Treg–like, and those with low and high CD45RO expressions were designated as naive-Treg–like and non-Treg–like clusters, respectively (Fig. 3E, 3F). Obviously, the effector-Treg–like cluster contained not only the cells that were identified as effector-Treg but also some of the FOXP3^low^ cells that were identified as non-Treg by manual gating (Fig. 3E, 3F). The HKK clustering was not computationally expensive: it took 45 s per patient, on average, to perform all three clustering steps using a conventional laptop.

#### Statistical comparisons of FOXP3^+^ T cell subpopulations between manual and automatic gating.

**FIGURE 4.**
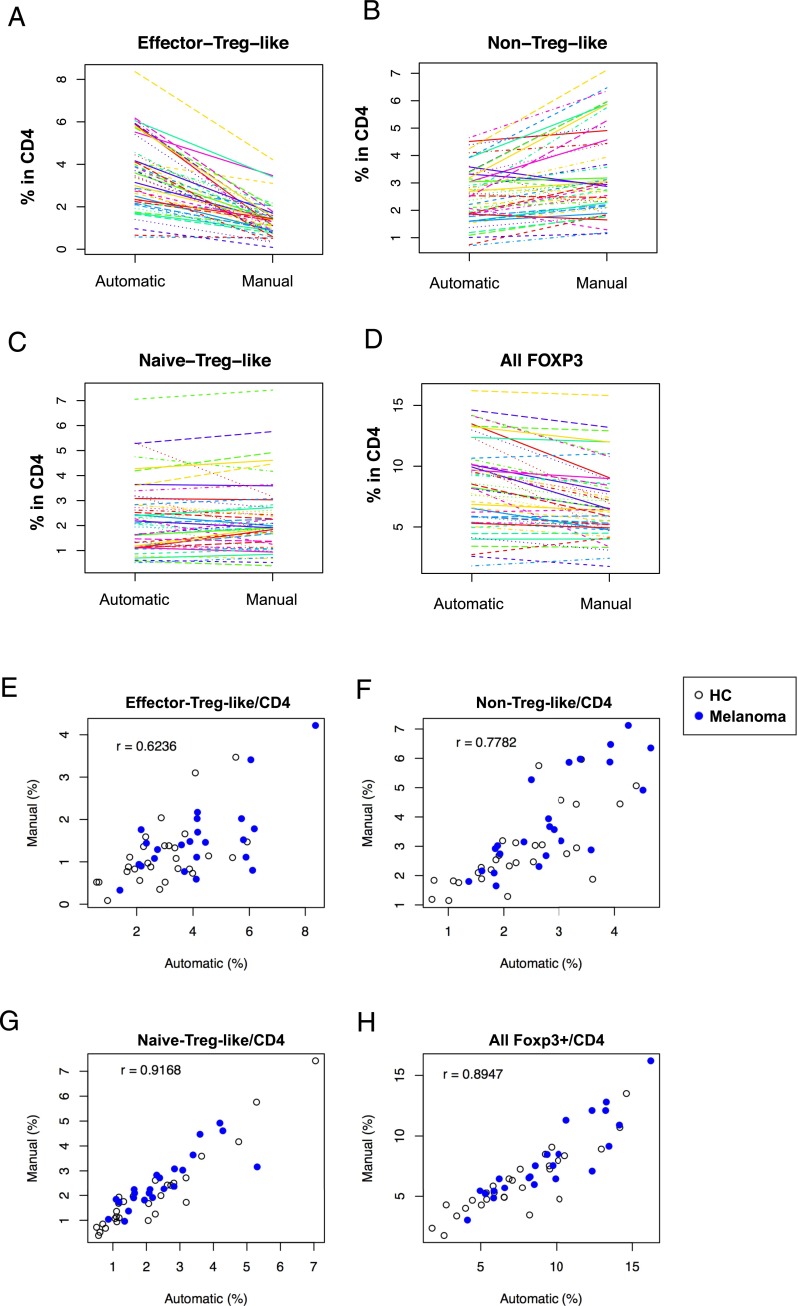
Comparison of the FOXP3^+^ T cell clusters/populations identified by the automatic and manual gating approaches. (**A**–**D**) The automatic (the HKK clustering) and manual gating approaches were compared by spaghetti plots of the percentage of the cells that were classified as (A) effector-Treg–like, (B) non-Treg–like, and (C) naive-Treg–like, and (D) all FOXP3^+^ T cells, in all samples including HCs and melanoma patients. (**E**–**H**) Scatterplots showing the percentages of each FOXP3**^+^** T cell cluster/population by automatic (the HKK clustering) and manual gating: (E) effector-Treg–like, (F) non-Treg–like, and (G) naive-Treg–like, and (H) all FOXP3^+^ T cells. Closed and open circles represent melanoma and HC samples, respectively. All percentages are in CD4^+^ T cells. Pearson correlation coefficient (*r*) was calculated using all the samples for each cluster.

We compared the subpopulations that were identified by the HKK clustering with those by the manual gating, to understand the similarities and dissimilarities of the two approaches. Obviously, the HKK clustering included more cells in the effector-Treg–like cluster than manual gating, whereas the latter included more in the non-Treg–like cluster (Fig. 4A–D). The effector-Treg–like and the non-Treg–like clusters showed relatively low correlations with their corresponding manually gated populations (*r* = 0.6236 and 0.7782, respectively; Fig. 4E, 4F). In contrast, the naive-Treg–like cluster and all FOXP3^+^ T cells had high correlations with their corresponding manually gated populations (*r* = 0.9168 and 0.8947, respectively; Fig. 4G, 4H). All results were statistically significant (*p* < 0.0001).

**FIGURE 5.**
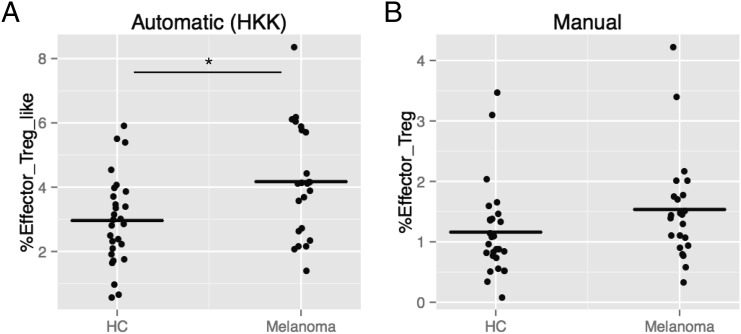
Effector-Treg–like cluster in HC and melanoma patients by the automatic HKK clustering or manual gating. (**A**) Box plot showing the percent effector-Treg–like/CD4 of HC and melanoma patients by the automatic HKK clustering. *Adjusted *p* < 0.05. (**B**) Box plot showing the percent effector-Treg/CD4 of HCs and melanoma patients by manual gating.

Next, the HKK clustering and the manual gating approaches were compared for association with melanoma. As we previously reported, the manually gated three subpopulations, effector-Treg, naive-Treg, and non-Treg, showed a significant difference between HCs and melanoma patients (*p* < 0.05) (8). However, assuming that the three subpopulations might be related to each other, when *p* values were adjusted for multiple comparisons using a Bonferroni method, all the adjusted *p* values exceeded 0.05; thus, the manually identified subpopulations did not show significant difference using the conservative approach. In contrast, among the automatically identified clusters, the effector-Treg–like cluster was significantly increased in melanoma patients compared with controls (adjusted *p* = 0.027; Fig. 5). This result suggested that the effector-Treg–like cluster by the HKK clustering more efficiently captured the characteristics of melanoma patients.

#### Application of automatic gating to an independent data set.

**FIGURE 6.**
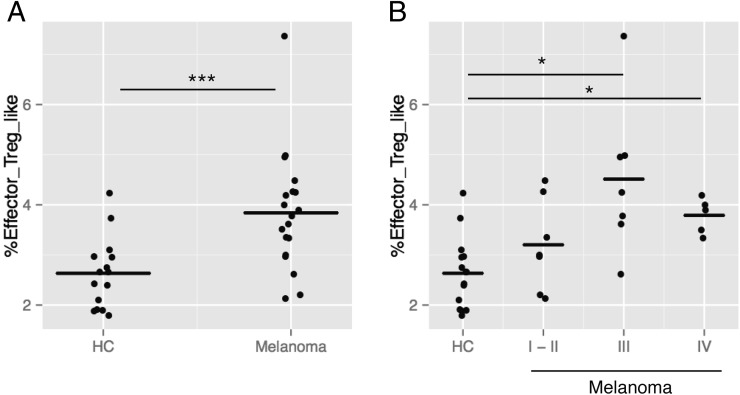
Application of the automatic gating approach to an independent data set. The established automatic gating method, the HKK clustering, was applied to an independent data set (the second data set, see Table I). (**A**) Box plots showing %(effector-Treg–like)/CD4 in HCs and melanoma patients (***adjusted *p* = 0.0015). (**B**) Box plots showing %(effector-Treg–like)/CD4 in HCs and different disease stages of melanoma patients (I-II, III, or IV). A Kruskal–Wallis test showed that the percentages were significantly different across different statuses (adjusted *p* = 0.0014). Pairwise comparisons were done by a Mann–Whitney–Wilcoxon test, showing significant differences (*adjusted *p* < 0.05).

Lastly, we attempted to apply the established method to an independent data set. We have generated a new data set using a different flow cytometer, *LSR Fortessa*, analyzing 12 HCs and 19 melanoma patients (the second data set; Table I). When *p* values were adjusted for the multiple comparisons of the three clusters, only the effector-Treg–like cluster showed a significant increase in melanoma patients compared with HCs by a Mann–Whitney–Wilcoxon test (adjusted *p* = 0.0015; Fig. 6), confirming the results of the first data set. Furthermore, a Kruskal–Wallis test showed that only the effector-Treg–like cluster was significantly different across HCs and three disease stages (adjusted *p* = 0.0014; Fig. 6A). Pairwise comparisons of the four different statuses using a Mann–Whitney–Wilcoxon test showed that the effector-Treg–like cluster was significantly increased in stages III and IV compared with HCs (adjusted *p* = 0.0127 and 0.0325, respectively; Fig. 6B).

**Table I.** Patient characteristics in the second data set

Patient No.	Age (y)/Sex	Type	Stage	TNM Classification	Previous Treatment
1	82/M	SSM	IA	pT1aN0M0	—
2	62/F	SSM	IB	T1bN0M0	—
3	52/M	SSM	IB	T2aN0M0	—
4	59/F	SSM	IB	pT2aN0M0	—
5	63/F	ALM	IIA	pT2bN0M0	—
6	60/M	ALM	IIC	pT4bN0M0	OP, Rec
7	78/F	SSM	IIC	T4bN0M0	—
8	64/M	SSM	IIIA	pT4N2aM0	—
9	53/F	SSM	IIIA	pT2aN1aM0	—
10	54/F	SSM	IIIB	pT1bN1aM0	OP, Rec
11	63/M	NM	IIIB	pT4bN1aM0	—
12	71/F	SSM	IIIB	T1aN1bM0	—
13	33/F	ALM	IIIB	pT3bN2aM0	—
14	78/M	ALM	IIIC	pT4bN3M0	—
15	71/M	ALM	IV	T4bN3M1c	OP, CT
16	62/M	MU	IV	pTxN0M1c	OP, CT
17	56/F	MU	IV	pTxN0M1c	OP, CT
18	39/F	SSM	IV	pTxNxM1c	OP, CT, RT
19	59/F	ALM	IV	TxN3M1c	OP

ALM, acral lentiginous melanoma; CT, chemotherapy; MU, mucosal melanoma; NM, nodular melanoma; OP, operation; Rec, recurrence; RT, radiotherapy; SSM, superficial spreading melanoma TNM, Tumor-Node-Metastasis.

## Discussion

This study has proposed to use a clustering approach to reveal the immunological profiles of FOXP3^+^ T cells without invoking the concept of the immunosuppressive phenotype of FOXP3^+^ T cells (19), and thereby to correlate them with disease phenotype or biological response. The current dogma of Tregs (i.e., the lineage perspective) (31) considers that a Treg population can be defined only when it has been shown to be unique and distinct from other T cells and stable as a lineage (19). To be compatible with the lineage perspective, the threshold level of FOXP3 for defining the effector-Treg subpopulation by manual gating has been determined based on the result of a suppressive assay (9), the gold standard for assessing the immunosuppressive function of Tregs (32, 33). Alarmingly, however, recent studies indicate that the suppressive activity by the assay is mostly explained by the absorption of IL-2 in the culture by CD25 (IL-2R α-chain) on the surface of anergic Treg (34, 35). Because FOXP3 has positive correlations with CD25 and anergy (36), it is not surprising that FOXP3^high^CD45RO^+^CD25^high^ T cells (i.e., effector-Treg by manual gating) show a high suppressive activity in the in vitro assay.

The proposed method revealed that the effector-Treg–like cluster was significantly increased only in melanoma patients in both of the data sets, suggesting that this cluster captured an important immunological feature. These results encourage the computational clustering approach to reveal the immunological features of T cells in tumor-bearing patients. In contrast, the computationally identified effector-Treg–like cluster included some memory-phonotype CD45RO^+^FOXP3^low^ non-Treg cells by manual gating (Fig. 3E, 3F, 4A–D) (8, 9). Although the result is difficult to be interpreted by the lineage perspective, our recently proposed model of Treg and FOXP3, the feedback control perspective (31), may be useful for reconciling the results in this study with findings on Tregs in the literature. Under this new perspective, the increase of the effector-Treg–like cluster in melanoma patients is interpreted as FOXP3 was more frequently induced in tumor-bearing patients as a consequence of Ag recognition and a negative feedback mechanism of T cell activation, which also explains the memory phenotype of the effector-Treg–like cluster. Interestingly, the second data set analysis showed that the effector-Treg–like cluster was increased in higher stages of melanoma (stages III and IV, Fig. 6), which is interpreted by the new model as reactive FOXP3^+^ cells accumulated in the immune system as a result of prolonged chronic stimulation by cancer cells. Although this study is not conclusive as to which perspective should be used, certainly the new perspective allows a more flexible interpretation of clinical and experimental data, because it does not assume stable and distinct lineages but is more concerned with the dynamics at the cellular and molecular levels. In fact, a recent study demonstrated an extensive TCR overlap between FOXP3^+^ cells and CD25^+^FOXP3^−^ cells at the site of inflammation (37), further confirming that T cells may dynamically change the expression of FOXP3, especially in disease conditions. Importantly, this view leads to a question whether the negative feedback mechanism is stronger than the positive feedback mechanism in melanoma patients, encouraging further investigations on the latter in future studies.

The proposed clustering method provided reproducible results between two independent data sets. Note that the first data set is in FCS2.0 format, obtained by an analog system, FACSCalibur, whereas the second data set is in FCS3.0 format, and was obtained by a digital acquisition system, LSR Fortessa (38). Although a data normalization method for such different data sets is yet to be established and is a big issue in flow cytometric data analysis (39), this study encourages the use of the proposed method or similar clustering methods for the analysis of complex flow cytometric data. In our analysis, CD4^+^ T cell selection was almost identical between the manual and the automatic approaches (Fig. 2A). Although the manual gating commonly creates a lymphocyte gate using FSC and SSC, and subsequently identifies CD4^+^ T cells (Fig. 1), our investigation showed that it was more efficient to identify CD4^+^ T cells by a one-step HDDC clustering approach using all FSC, SSC, and CD4. This indicates that CD4^+^ T cells are the most distinct when all three dimensions are used. In fact, it is a common practice in manual gating to use either FSC or SSC to create a CD4-gate (e.g., Fig. 1C). This result confirms that flow cytometric data analysis in a higher dimensional space enables more efficient analysis (23, 25), which is widely accepted but yet to be further demonstrated by addressing real immunological problems. In contrast, there is a small discordance in FOXP3^+^ T cells by the manual gating and by the automatic clustering (Fig. 2B, *left panel*), although the overall correlation was high (*r* = 0.8947; Fig. 4H). This small discordance may be related to either or both of the inherent arbitrariness of the manual gating approach to the FOXP3^+^ selection and the variations by k-means. Because FOXP3 is measured by intranuclear staining and, therefore, its autofluorescence background is high (Fig. 1) (9, 40), it is experimentally difficult to precisely determine the boundary between the negative cloud and positive cells without using FOXP3-deficient T cells from mutant humans (41), which are practically difficult to be obtained in most laboratories. In addition, even using such negative controls, it is not obvious where to set the boundary. This is why the manual gating approach visually identifies a groove between the negative cloud and positive cells and sets it as a boundary for FOXP3^+^ cells (Fig. 1D). In contrast, k-means is a method to determine clusters by minimizing the within-cluster sum of squares (42), and thus can be affected by the distribution of cells and also by outliers, which can introduce variations. The small discordance of the boundary for FOXP3^+^ cells may have contributed to those of the non-Treg–like and naive-Treg–like clusters as well, because both clusters faced the boundary of FOXP3^+^ and FOXP3^−^ cells, whereas it presumably did not directly affect the effector-Treg–like cluster (Fig. 3E). However, considering that the proposed automatic gating identified a significant increase of only the FOXP3^+^ T cell subpopulation in melanoma patients, and that the manual gating did not produce any significant results on the analyzed subpopulations including non-Tregs and naive-Tregs, the immunological feature of melanoma patients most probably is in the cells with higher FOXP3 expressions, and the discordance in the FOXP3 boundary is probably not important in the setting. Yet, it is hoped that future studies will develop data analytic and modeling methods to better deal with the problem of where to set the boundary between negative and positive cells in continuous distributions.
